# Helminths found in common species of the herpetofauna in Ukraine

**DOI:** 10.3897/BDJ.12.e113770

**Published:** 2024-01-16

**Authors:** Oleksii Marushchak, Yaroslav Syrota, Ivanna Dmytrieva, Yuri Kuzmin, Andrii Nechai, Olga Lisitsyna, Roman Svitin

**Affiliations:** 1 I. I. Schmalhausen Institute of Zoology of National Academy of Sciences of Ukraine, Kyiv, Ukraine I. I. Schmalhausen Institute of Zoology of National Academy of Sciences of Ukraine Kyiv Ukraine; 2 Université de Strasbourg, CNRS, IPHC UMR7178, Strasbourg, France Université de Strasbourg, CNRS, IPHC UMR7178 Strasbourg France; 3 Institute of Parasitology, Slovak Academy of Sciences, Košice, Slovakia Institute of Parasitology, Slovak Academy of Sciences Košice Slovakia; 4 African Amphibian Conservation Research Group, Unit for Environmental Sciences and Management, North-West University, Potchefstroom, South Africa African Amphibian Conservation Research Group, Unit for Environmental Sciences and Management, North-West University Potchefstroom South Africa; 5 Educational and Scientific Institute of High Technologies, Taras Shevchenko National University of Kyiv, Kyiv, Ukraine Educational and Scientific Institute of High Technologies, Taras Shevchenko National University of Kyiv Kyiv Ukraine

**Keywords:** endoparasites, biodiversity, herpetofauna, common species, helminths, parasitic worms, geocoded occurrence

## Abstract

**Background:**

Only a few comprehensive studies have been carried out on parasites in amphibians and reptiles in Ukraine. This has resulted in identifying over 100 helminth species across these vertebrate groups. However, most of the studies were performed in the 20^th^ century and the taxonomy of many parasites and their hosts has changed ever since, in addition to the discovery of new species and registrations of species that had not been previously known for Ukraine. In recent decades, there have been very few publications on helminths from amphibian or reptile hosts in this region. Notably, just one of these recent studies is a faunistic study, providing a list of helminths found in two species of green frogs – *Pelophylaxridibundus* (Pallas, 1771) and *Pelophylaxesculentus* (Linnaeus, 1758). Therefore, it is clear that publishing datasets of modern records of helminths in these vertebrate groups, based on modern taxonomy, is an essential step in further studies of their parasitic diversity. Additionally, such study is important in terms of global climate change, the growing number of possibilities of invasion of alien species (both hosts and parasites) that might potentially become a threat to native biota and growing anthropogenic pressure on local populations of hosts that affect the parasites as well. In future, this study is planned to be used for the creation of a checklist of helminths of the herpetofauna of Ukraine. The present dataset is an inventory of various species of helminths parasitising common species of the herpetofauna in central, northern, western and southern Ukraine recorded during field studies in the 2021-2023 period.

**New information:**

The dataset is the first one to represent the up-to-date and unified data on helminths of reptiles and amphibians of Ukraine. Previously, records of this group of organisms with reference to their hosts were presented as several separate records within the country. Currently, this is the largest dataset presenting geocoded records of non-human-related helminths in the fauna of Ukraine. It reports helminth species from 15 hosts (205 individuals), including eight amphibians and seven reptilian species found in various Ukrainian regions. A total of 47 helminth species have been documented in the research and during 2021-2023 period on the territory of northern (Kyiv and Zhytomyr), western (Lviv, Zakarpattia Ivano-Frankivsk), central (Vinnytsia, Dnipropetrovsk, Cherkasy, Zaporizhzhia and Poltava) and southern (Odesa) regions of Ukraine. The identified helminth species belong to the following phyla: Acanthocephala (Centrorhynchidae (2), Echinorhynchidae (2)); Nematoda (Acuariidae, Anisakidae, Cosmocercidae (3), Dioctophymatidae, Gnathostomatidae (1), Kathlanidae (1), Molineidae (7), Onchocercidae (1), Pharyngodonidae (1), Rhabdiasidae (6), Strongyloididae); Platyhelminthes (Diplodiscidae (1), Diplostomidae (2), Encyclometridae (1), Haematoloechidae (1), Leptophallidae (2), Macroderidae (1), Mesocestoididae, Opisthorchiidae (2), Plagiorchiidae (3), Pleurogenidae (2), Polystomatidae (3), Proteocephalidae (1), Strigeidae (1) and Telorchiidae (3)). Only some helminths in the dataset were not identified to species level. Material is stored in the collection of the department of Parasitology of the I. I. Schmalhausen Institute of Zoology NAS of Ukraine.

## Introduction

According to the known literature (Lindenmayer et al. 2011, Koprivnikar et al. 2011, Kopecký et al. 2013, Vasyliuk et al. 2015, García‐Díaz et al. 2016, Demkowska-Kutrzepa et al. 2018, Nekrasova et al. 2019, Marushchak et al. 2021), amphibians are currently the most threatened class of vertebrate animals and nearly a third of amphibian species are already extinct or are on the edge of extinction due to critical decline of their populations. Amphibians play an important role in food chains, serving as predators and prey for many other animals. This characteristic is related to the infection of amphibians with various parasites of larval and adult stages. This makes studying amphibian parasites a valuable tool for ecological studies, as the intensity and prevalence of infection, i.e. the abundance and occurrence of parasites can reflect the state of the whole ecosystem. Reptiles often serve as apex predators (e.g. monitors) or tertiary consumers (e.g. snakes) and rarely occupy lower positions in the food chains (Lindeman 1942, Bannikov et al. 1977). Nevertheless, reptiles often share habitats with amphibians and frequently harbour closely-related parasite species, making them important for parasitological studies. Moreover, both amphibians and reptiles are known to have limited capacity for long-distance migrations (Bannikov et al. 1977, Kuzmin 2012). Thus, it makes their parasitofauna suitable for representing the current state of separate local parts of the ecosystem and interactions between them, too (Okulewicz et al. 2014, Kuzmin et al. 2020, Čeirāns et al. 2021, Čeirāns et al. 2023). Particular interest in recent decade was drawn to studies of parasitofauna of those exotic (therefore potentially invasive) amphibians and reptiles kept in terrariums that might potentially become sources of invasive species of helminths to native herpetofauna of Ukraine (Kostko and Barkar 2019, Stets 2019).

Several significant studies dealing with terrestrial cold-blooded vertebrate parasites were performed on Ukraine's territory in the 20^th^ century. Mazurmovich (1951) and Maguza (1973) investigated helminths of amphibians in the northern part of Ukraine. The study of Mazurmovich (1951) focused on the fauna of frogs' helminths, their life cycles and their impact on their hosts. A subsequent study by Maguza (1973) mainly focused on the helminths fauna and morphology of species. Unfortunately, at the time, the taxonomy of helminths, as well as some frogs (e.g. *P.esculentus* was not recognised) was incomplete and many of the species identifications (especially trematodes and nematodes) in both studies were incorrect. Later on, Ryzhikov et al. (1980) summarised all available data on amphibian helminths from broad territories of the former USSR. In their work, the Ukrainian species of amphibians were hosts to a total of 92 species of helminths (23 in urodelans and 90 in anurans), which included 41 species of trematodes, 37 nematodes, eight acanthocephalans, three cestodes and two monogeneans. During the same period, only scattered findings of helminths from reptiles in Ukraine were published (Ivanitskiy 1927, Vlasenko 1930, Modrzejewska 1938, Ivanitskiy 1940). Nonetheless, a monumental study by Sharpilo (1976) comprehensively compiled all the data available at that time on reptiles from the former USSR and included information on about 100 species of helminths parasitising snakes, lizards and turtles in Ukraine. These species included around 35 trematodes, around 50 nematodes, six cestodes, five acanthocephalans and one monogenean. Many of these species were found in larval stages (thus frequently identified to the genus or higher level), frequently sharing amphibian and reptile host species. Both latter studies included the identification keys and descriptions of species of trematodes, nematodes, cestodes, monogeneans and acanthocephalans. At the beginning of the 21^st^ century, no all-encompassing studies of helminths on the territory of Ukraine took place. All research was focused mainly on separate morphological, ecological and biological characteristics of individual species or groups of helminths (Kuzmin 1993, Kuzmin 2000, Kuzmin et al. 2020, Kuzmin et al. 2023) or helminthofauna of particular host species from different locations (e.g. helminths of Ranidae frogs from north-western part of the Polissia Region of Ukraine (Kuzmin et al. 2012) or description of *Hexametraquadricornis* (Wedl, 1861) from the Crimean Peninsula (Kuzmin and Kukushkin 2012). Even if the territory of Ukraine had been studied in general, it appeared to be only a part included in a much larger geographical area, like Palearctica in the summarising study of helminths of sand lizards (Sharpilo et al. 2001).

In total, over 100 different helminth species were recorded from amphibians and reptiles in Ukraine, nearly a third of which were registered in the larval stage (using herpetofauna as intermediate or paratenic hosts) (Sharpilo 1976, Ryzhikov et al. 1980). However, the systematics of these parasites have undergone major changes (for example, nematodes were referred to as Class in both studies) and some species were described from studied hosts in Europe (and Ukraine as well): (e.g. *Oswaldocruzialisnykiensis* Svitin, 2017, described from a forest massif near Kyiv, Ukraine by Svitin (2017)); *Rhabdiasesculentarum* Cipriani, Mattiucci, Paoletti, Santoro & Nascetti, 2012, described in Europe by Cipriani et al. (2012)); synonymised (e.g. *Oswaldocruziagoezei* Skrjabin & Schultz, 1952 (synonym of *Oswaldocruziafiliformis* Goeze, 1782), *Hexadontophorus* spp., *Paraentomelas* spp.) or reinstated (e.g. *Oswaldocruziaskrjabini* Travassos, 1917) since the mentioned studies were published (Baker 1980, Baker 1987, Cipriani et al. 2012, Svitin 2015, Svitin 2017). Moreover, several host species were re-identified within their known natural range (e.g. *Anguiscolchica* (Nordmann, 1840) (Jablonski et al. 2021) and *Hylaorientalis* Bedriaga, 1890 (Manilo et al. 2014) have not been known in Ukraine at that time), as well as parasite species being synonymised or renamed. The only recent study dealing with helminths of Ukrainian amphibians with up-to-date systematic included 19 species of trematodes, seven species of nematodes and one acanthocephalan collected from frogs *P.esculentus* and *P.ridibundus* (Kuzmin et al. 2020). Therefore, the new checklist, based on currently-recognised parasite species from different Ukrainian amphibians (currently 22 known species: Anura - 15, Caudata - 7) and reptiles (currently 24 known species: Testudines - 1, Squamata - 23) (Bannikov et al. 1977, Kuzmin 2012, Manilo et al. 2014, Jablonski et al. 2021), will be helpful for further ecological studies in the area.

## General description

### Purpose

The primary objective of this study is to investigate helminth diversity in the predominant species of amphibians and reptiles in Ukraine, except for those listed in the Red Data Book of Ukraine. Accordingly, we reduced our sampling efforts when the likelihood of discovering additional helminth species within specific hosts became low. Additionally, we did not sample many host species that do not typically have specific helminths. For instance, after studying 23 specimens of *Ranatemporaria* Linnaeus, 1758, we only dissected three specimens of *Ranaarvalis* (Nilsson, 1842) because both frog species usually share the same helminth species (Ryzhikov et al. 1980).

Of all studied host species, only the common newt did not have any helminths detected (however, it should be noted that only one specimen was examined). While the common newt is not listed as an endangered species, we could not locate an area with a dense population of this species. According to literature data (Ryzhikov et al. 1980), the common newt has no specific helminth parasites. Due to that, we chose to limit the dissection of this host to avoid putting additional pressure on its populations.

### Additional information

In the study, we collected a total of 47 species of helminths. That includes nearly all common parasite species, some of which have not been documented previously for the territory of Ukraine. For instance, two specimens of the acanthocephalan *A.ranae* were obtained from the intestine of the European pond turtle. Both were found attached, suggesting that the turtle most likely became infected by ingesting their intermediate host, *Asellusaquaticus* (Linnaeus, 1758) (Malacostraca, Asellidae). Additionally, for the first time, we recorded larvae of two distinct *Mesocestoides* species in the body cavity of common vipers. Notably, only one of them, namely *Mesocestoideslineatus* Goeze, 1782, had been previously reported in Ukrainian reptiles (Sharpilo 1976).

In our study, we tried to identify all parasites at the species level. However, we frequently encountered metacercariae and cystacanths in amphibians and early-stage nematode larvae in both amphibians and reptiles. These helminth specimens could only be identified to the family, class or phylum level. Amongst these records (n = 48) identified to a higher-than-specific level, there are 13 records identified to the level of genus (*Oswaldocruzia* - 5, *Eustrongylides* - 1, *Strongyloides* - 1, *Neyraplectana* - 1, *Contracaecum* - 2, *Tylodelphys* - 1, *Mesocestoides* - 2), 22 records - to the level of family (Strigeidae - 9, Cosmocercidae - 9, Acuariidae - 2, Plagiorchiidae - 1, Centrorhynchidae - 1), five records - to the level of superfamily (Metastrongyloidea - 5), two records - to the level of order (Spirurida - 2), one record - to the level of class (Trematoda - 1) and five records - to the level of phylum (Nematoda - 5). We believe that molecular methods can assist in their identification in the future, assuming data on adult parasites are deposited in GenBank.

The dataset presented provides current information on parasite species found in regions of Ukraine that are readily accessible. In addition to a list of parasites, the data encompasses their geographical location, site of infection and infection intensity (Svitin et al. 2023). While exploring other regions could potentially reveal more parasite species, we believe this dataset offers a valuable background for further research, including analyses of parasite communities amongst the examined hosts and comparative studies of other hosts and in other regions.

## Project description

### Title

Creating the genetic database of helminths from common species of amphibians and reptiles from the territory of Ukraine

### Personnel

Yaroslav Syrota (Point of contact)

### Study area description

The project covers different territories of Ukraine and includes the invasive study of communities of helminths presented in the target studied species of herpetofauna.

The aims of the projects include:

- collecting material from the maximum possible number of species of amphibians and reptiles from different regions of Ukraine;
- identifying the collected parasites using light microscopy methods;
- obtaining the genetic sequences of the most widely used nuclear (18S) and mitochondrial (cox1) markers.

### Funding

The project is mainly funded by the Grant for Young Scientists (0123U100296) of the National Academy of Sciences of Ukraine and is performed in the I. I. Schmalhausen Institute of Zoology NAS of Ukraine. The publishing of the study is supported by the EU 508 NextGenerationEU through the Recovery and Resilience Plan for Slovakia under projects No. 09I03-03-V01-00046 and No. 09I03-03-V01-00016. Additionally, the research was partly funded by the project Emys-R https://emysr.cnrs.fr through the 2020-2021 Biodiversa & Water JPI joint call for research proposals, under the BiodivRestore ERA-Net COFUND programme and with the funding organisations Agence Nationale de la Recherche (ANR, France), Bundesministerium für Bildung und Forschung (BMBF, Germany), State Education Development Agency (VIAA, Latvia) and National Science Center (NSC, Poland).

## Sampling methods

### Study extent

The authors of this project collected 440 records of helminths, of which 48 records were identified only to higher taxonomic categories of parasites from common species of amphibians and reptiles from various localities within Ukraine. All species were studied by light microscopy methods and the geocoded dataset was created. The presented dataset expands the knowledge on the recorded helminths' species of amphibians and reptiles, as there had been less than 50 records of these representatives of the world's fauna in Ukraine known before the dataset was published.

### Sampling description

**Figure 1.**
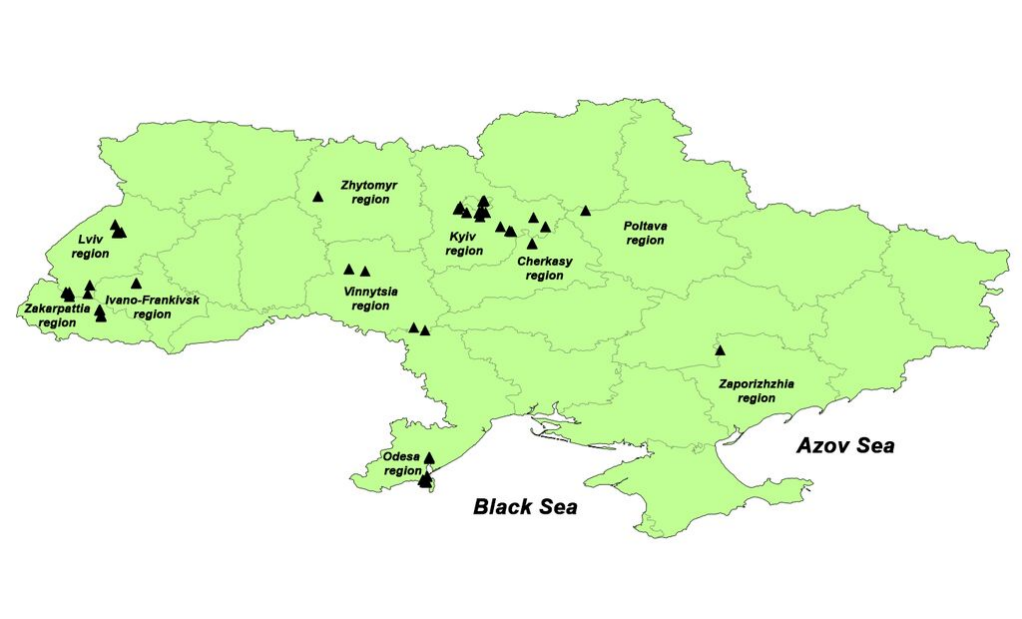
Map of records of helminths of herpetofauna on the territory of Ukraine.

During the reporting period, several field trips were performed to various regions of Ukraine in order to collect material. The most favourable period for collecting amphibians is spring when they gather in large groups during spawning. However, due to security reasons as a result of the current Russian aggression, most of the hosts for this project were collected in late April and early May and until the beginning of November. This period is not critical since the level of helminth infection of the hosts increases due to active feeding, movement and completion of the spawning period (parasites that have entered the bodies of the hosts in the early larval stages had enough time to develop to adult stages) compared to the period immediately after wintering and at the time of spawning, when the hosts hardly eat, while the individuals weakened by hyperinfection of parasites died during wintering. During the warm periods of 2021, 2022 and 2023, a total of 205 amphibians and reptiles (Marushchak et al. 2023) from Zakarpattia, Lviv, Ivano-Frankivsk, Poltava, Zhytomyr, Vinnytsia, Odesa, Cherkasy and Kyiv Regions and the City of Kyiv (Fig. 1) were captured and examined. These hosts included anuran amphibians: 23 grass frogs *R.temporaria*, three moor frogs *R.arvalis*, 12 common toads *Bufobufo* (Linnaeus, 1758), 22 green toads *Bufotesviridis* (Laurenti, 1768), 12 common spadefoots *Pelobatesfuscus* (Laurenti, 1768), six marsh frogs *P.ridibundus*, five eastern tree frogs *H.orientalis*, seven European fire-bellied toads *Bombinabombina* (Linnaeus, 1761) and one common newt *Lissotritonvulgaris* (Linnaeus, 1758); and reptiles: 23 sand lizards *Lacertaagilis* Linnaeus, 1758, 21 viviparous lizards *Zootocavivipara* (Lichtenstein, 1823), six eastern slowworms *A.colchica*, 23 grass snakes *Natrixnatrix* (Linnaeus, 1758), seven dice snakes *Natrixtessellata* (Laurenti, 1768), 27 European pond turtles *Emysorbicularis* (Linnaeus, 1758) and seven common vipers *Viperaberus* (Linnaeus, 1758). Most amphibians and reptiles were caught by hand or with a net (using a modified hydrobiological net or strong fishing net) and several terrapins were captured in traps. The turtle trap used was the modified crab trap with wider openings and an attached plastic container for the bait. The most effective bait was freshly euthanised fish caught in the same water, though spoiled fish and the visceral organs of other turtles were successfully used.

For visualising the records, the points of herpetofauna registrations were collected (with the indication of latitude 00.00000 N and longitude 00.00000 E) using the field off-line orientation programme MAPS.ME (version 12.0.1-Google) and Google Earth Pro (version 7.3.3). Visualisation of records and creation of maps was carried out in the QGIS program (v.2.181, QGIS Development Team, 2016. QGIS Geographic Information System. Open Source Geospatial Foundation. URL http://qgis.org). Species were identified using methodological materials (Bannikov et al. 1977, Nekrasova et al. 2005, Kuzmin 2012).

The dataset visualisation was conducted using the R programming language (R core Team 2023). Initially, the dataset containing information about various hosts was merged with the parasites' dataset. Following this, parasitological metrics, such as total prevalence, richness, mean intensity and host sample size, were computed for each host species utilising versatile functions from the *dplyr* package. After that, the obtained data were visualised using the *ggplot2* package.

Autopsies were performed in field laboratories during expedition trips. Otherwise live animals were transported to the laboratory of the I. I. Schmalhausen Institute of Zoology, National Academy of Sciences of Ukraine. Plastic containers of suitable sizes with holes for ventilation lined with moist plant substrate, paper towels or soft cloth were used to transport the hosts. Amphibians and reptiles were euthanised by injecting 10% lidocaine in the brain or bloodstream (only terrapins) and examined for helminths. In amphibians, the spinal cord was cut and the canal was flushed with saline using a thin Pasteur pipette to detect trematode larvae.This could not be done for reptile parasites due to a thin spinal canal that is too narrow and long. All organs were removed, washed in saline and checked for parasites. The body cavity, body and limb muscles and subcutaneous space were also checked for parasites in selected individuals. Found parasites were carefully removed, placed in small Petri dishes with saline and then fixed accordingly to the taxonomic group. Nematodes and smaller trematodes were fixed with hot 70% ethanol; monogeneans, large trematodes and cestodes were euthanised with hot water and then fixed with 70% ethanol; acanthocephalans were left in distilled water for up to 24 hours for the proboscis to extend and then fixed with 70% ethanol. For larval stages found in cysts, some specimens were removed using forceps and needles and fixed with 70% ethanol, other specimens were fixed in cysts or (in case of hyperinfection with a single species) counted and only a sample was taken. All parasites were transferred to vials with 70% ethanol and subsequently stored in the fridge. Several monogeneans were fixed in 10% formalin and stored at room temperature.

For the microscopic studies, nematodes and smaller trematodes were placed in distilled water for about 20-60 min, cleared in lactophenol for 30-120 min and studied as temporary mounts. Larger trematodes were stained with Mayer's haematoxylin in the following stages: soaking the material preserved in 70% ethanol in water (5 min); immersion in dye (5–10 min depending on the size of the individuals); immersion in a solution of hydrochloric acid (5 min); ammonia solution (5 min); passing through alcohols of increasing concentration (70%, 80%, 90%, 96%, 100% – 5(7) min for each stage); cleared in clove oil (10–15 min) and inclusion in Canadian balm on a permanent slide.

Cestodes were stained with carmine according to the following scheme: immersion in dye (5–10 min); acidified alcohol (5 min); passing through alcohols of increasing concentration (80%, 90%, 96%, 100% - 5(7) min for each stage); clearing in clove oil (10–15 min) and mounted in Canada balm on a permanent slide.

A study on the morphology of helminths was performed under an AmScope T690B light microscope with a digital camera; photomicrographs were obtained on a ZEISS Axio Imager M1 System microscope at the Center for Collective Use of Scientific Instruments "Animalia" (https://www.izan.kiev.ua/cen-coll.htm) at the Institute of Zoology. The image plate was composed using Photoshop (CS5 v.12.1.0) software. The original descriptions and latest redescriptions of species were used for the identification, as well as the identification keys in the monographs by Ryzhikov et al. (1980) and Sharpilo and Iskova (1989) and in separate articles (Svitin and Kuzmin 2012, Kuzmin and Kukushkin 2012, Svitin 2017). Some individuals of the larval stages of nematodes and trematodes were not identified at the species level due to the lack of diagnostic features.

### Quality control

The authors of the dataset are fully responsible for the quality of data provided: accuracy of identification, counting etc.

### Step description

1. Conducting field trips in search of the hosts;
2. Collecting the living hosts by hand, traps or net and field identification of their species;
3. Autopsies of the hosts, dissections and study of helminths;
4. Extracting and identification of helminths according to the standard methods;
5. Georeferencing;
6. Organising the dataset according to the Darwin Core standards.

## Geographic coverage

### Description

The dataset represents the records of helminths made on the territory of Ukraine, namely those regions that could be reached by the field expeditions under the conditions of war: Zaporizhzhia, Zhytomyr, Lviv, Vinnytsia, Cherkasy, Zakarpattia, Ivano-Frankivsk, Poltava, Odesa, Dnipropetrovsk and Kyiv administrative regions.

### Coordinates

44.402 and 52.376 Latitude; 22.17 and 40.188 Longitude.

## Taxonomic coverage

### Description

**Figure 2.**
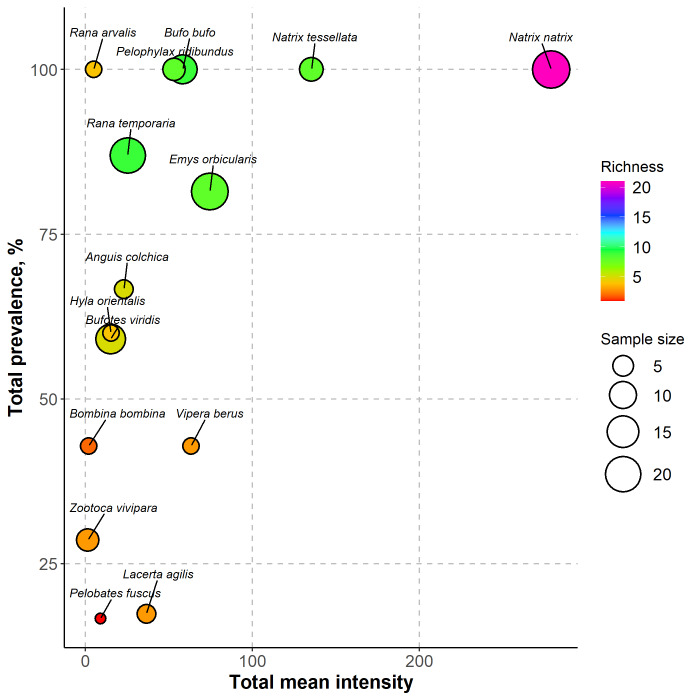
Visualising the helminth community of common herpetofauna species in Ukraine. Each point represents a helminth component community of a host species. The x-axis shows the prevalence of infection, which is the proportion of hosts infected with at least one helminth species. The y-axis shows the mean intensity of infection, which is the average number of helminth individuals per infected host. The colour of the points indicates the richness of the helminth community, which is the number of helminth species found in each host. The size of the points shows the host sample size.

**Figure 3.**
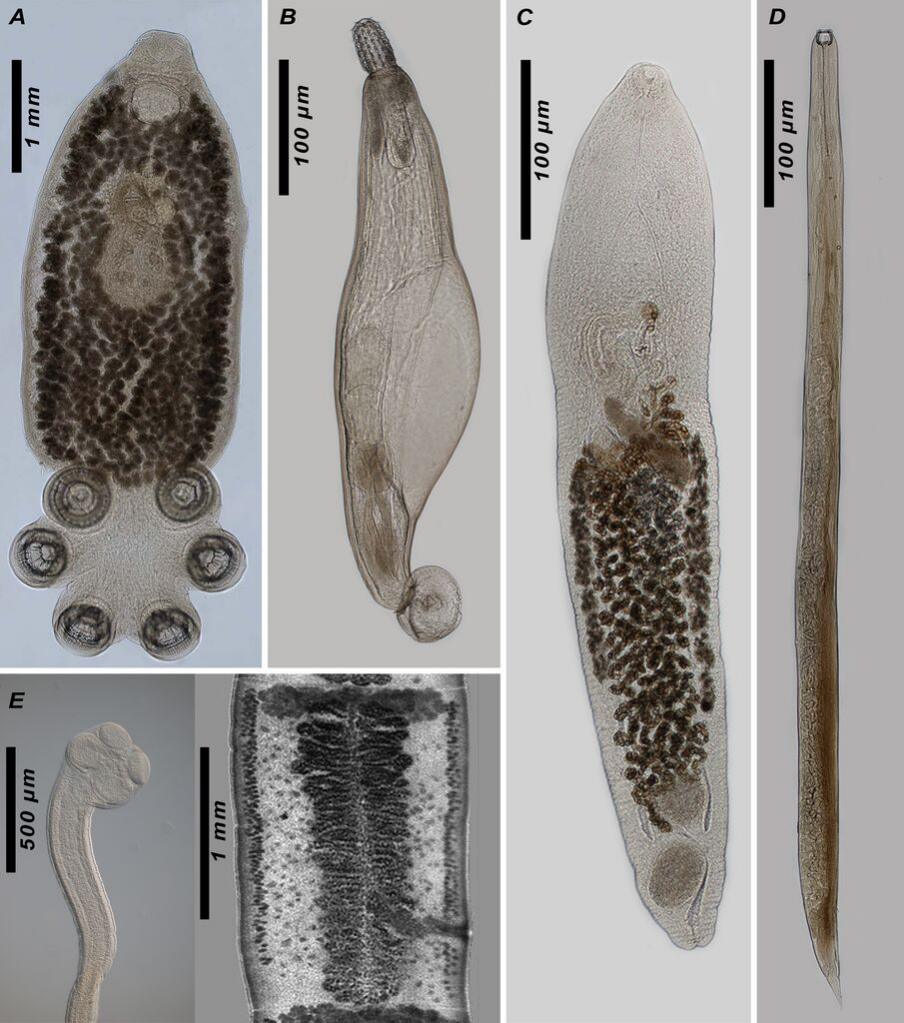
Representatives of the major taxa (Monogenea, Acanthocephala, Trematoda, Nematoda and Cestoda) found in the present study: **A** *Polystomoidesocellatum*; **B** *Pseudoacanthocephalusbufonis*; **C** *Telorchisstossichi*; **D** *Entomelasentomelas*; **E** *Ophiotaeniaeuropaea*.

The dataset consists of the records of helminths from the three most represented phyla: Nematoda, Acanthocephala and Platyhelminthes. It represents the findings of 47 different helminth species from 15 different hosts (8 from class Amphibia and 7 from class Reptilia (Fig. 2)) from different areas of Ukraine. Fourteen remaining species of amphibians and 17 species of reptiles were not included in the study because of their conservation status or the presence of their populations within the occupied territories where the authors had no access. Some helminths were identified only to the higher taxonomic levels: seven genera, five families, one superfamily, two orders, one class and one phylum (Fig. 3, Table 1).

### Taxa included

**Table:** 

Rank	Scientific Name	
kingdom	Animalia	
phylum	Acanthocephala	
class	Palaeacanthocephala	
order	Echinorhynchida	
family	Echinorhynchidae	
order	Polymorphida	
family	Centrorhynchidae	
phylum	Nematoda	
class	Chromadorea	
order	Dioctophymatida	
family	Dioctophymatidae	
order	Rhabditida	
family	Acuariidae	
family	Anisakidae	
family	Cosmocercidae	
family	Gnathostomatidae	
family	Kathlanidae	
family	Molineidae	
family	Onchocercidae	
family	Pharyngodonidae	
family	Rhabdiasidae	
superfamily	Metastrongyloidea	
family	Strongyloididae	
order	Spirurida	
phylum	Platyhelminthes	
class	Cestoda	
order	Cyclophyllidea	
family	Mesocestoididae	
order	Onchoproteocephalidea	
family	Proteocephalidae	
class	Monogenea	
order	Polystomatidea	
family	Polystomatidae	
class	Trematoda	
order	Diplostomida	
family	Diplostomidae	
family	Strigeidae	
order	Plagiorchiida	
family	Diplodiscidae	
family	Encyclometridae	
family	Haematoloechidae	
family	Leptophallidae	
family	Macroderidae	
family	Opisthorchiidae	
family	Plagiorchiidae	
family	Pleurogenidae	
family	Telorchiidae	

## Temporal coverage

**Formation period:** 2021/2023.

### Notes

The parasites' hosts were collected during the vegetation period, when the hosts began to be active, to the period of seasonal decreasing of the activity: from March to October.

## Usage licence

### Usage licence

Creative Commons Public Domain Waiver (CC-Zero)

## Data resources

### Data package title

Particular records of helminths from common species of herpetofauna of Ukraine

### Resource link

https://doi.org/10.15468/v45tya

### Alternative identifiers

https://www.gbif.org/uk/dataset/ec73e150-38ce-46ff-9268-70bc0dd86a60

### Number of data sets

1

### Data set 1.

#### Data set name

Particular records of helminths from common species of herpetofauna of Ukraine

#### Data format

Darwin Core

#### Data format version

1.8

#### Description

The dataset consists of 440 records of helminths belonging to 47 species; 48 records were identified only to higher taxonomic categories. A total of 11305 parasites individuals were recorded from 205 hosts belonging to eight amphibian and seven reptile species. Such stages as mature individuals, larvae and cysts were taken into account after examining such parts of the hosts' bodies as muscles, stomach, liver, lungs, body cavity, intestine, rectum, spine, pharynx, oesophagus and bladder (Svitin et al. 2023).

The dataset presented provides current information on parasite species found in regions of Ukraine that are readily accessible. In addition to a list of parasites, the data encompasses their geographical location, site of infection and infection intensity. While exploring other regions could potentially reveal more parasite species, we believe this dataset offers a valuable background for further research, including analyses of parasite communities amongst the examined hosts and comparative studies with other hosts and regions.

**Data set 1.** 

Column label	Column description
occurrenceID	http://rs.tdwg.org/dwc/terms/occurrenceID; a unique identifier of a particular occurrence within this dataset.
scientificName	http://rs.tdwg.org/dwc/terms/scientificName; the full scientific name, with authorship and date information, if known, of the identified species or other taxonomic level.
basisOfRecord	http://rs.tdwg.org/dwc/terms/basisOfRecord; a specific nature of the way in which the data were recorded.
eventDate	http://rs.tdwg.org/dwc/terms/eventDate; the date-time or interval during which the observation was made.
verbatimeventDate	http://rs.tdwg.org/dwc/terms/verbatimEventDate; an original version of the recorded date-time or interval during which the observation was made.
taxonRank	http://rs.tdwg.org/dwc/terms/taxonRank; the taxonomic rank of the record made.
decimalLatitude	http://rs.tdwg.org/dwc/terms/decimalLatitude; the geographic latitude (in decimal degrees, using the spatial reference system given in dwc:geodeticDatum) of the geographic centre of a location, where the observed individual (its host actually) was spotted.
decimalLongitude	http://rs.tdwg.org/dwc/terms/decimalLongitude; the geographic longitude (in decimal degrees, using the spatial reference system given in dwc:geodeticDatum) of the geographic centre of a location, where the observed individual (its host actually) was spotted.
coordinatesUncertaintyInMetres	http://rs.tdwg.org/dwc/terms/coordinateUncertaintyInMeters; the horizontal distance (in metres) from the given latitude and longitude describing the smallest circle containing the whole of the location.
geodeticDatum	http://rs.tdwg.org/dwc/terms/geodeticDatum; the ellipsoid, geodetic datum or spatial reference system (SRS), upon which the geographic coordinates given in decimalLatitude and decimalLongitude are based.
language	http://purl.org/dc/terms/language; a language of the resource.
taxonRemarks	http://rs.tdwg.org/dwc/terms/taxonRemarks; comments or notes about the taxon or name.
organismRemarks	http://rs.tdwg.org/dwc/terms/organismRemarks; comments or notes about the registered organism(s).
occurrenceRemarks	http://rs.tdwg.org/dwc/terms/occurrenceRemarks; comments or notes about the occurrence of the organism. In this dataset - localisation of the helminths in different parts of the host's body.
organismQUantity	http://rs.tdwg.org/dwc/terms/organismQuantity; a number or enumeration value for the quantity of the registered organisms.
organismQuantityType	http://rs.tdwg.org/dwc/iri/organismQuantityType; the type of quantification system used for the quantity of organisms.
georeferenceProtocol	http://rs.tdwg.org/dwc/terms/georeferenceProtocol; a description or reference to the methods used to determine the spatial footprint, coordinates and uncertainties.
recordedBy	http://rs.tdwg.org/dwc/iri/recordedBy; a person, group or organisation responsible for recording the original registration of the species.
identifiedBy	http://rs.tdwg.org/dwc/terms/identifiedBy; a list (concatenated and separated) of names of people, groups or organisations who assigned the taxon to the registered organism.
georeferencedBy	http://rs.tdwg.org/dwc/iri/georeferencedBy; a person, group or organisation who determined the georeference (spatial representation) for the location where the host of the parasite(s) was caught.
materialSampleID	http://rs.tdwg.org/dwc/terms/materialSampleID; an identifier for the sampled hosts, where the parasites were found.
associatedTaxa	http://rs.tdwg.org/dwc/terms/associatedTaxa; a list (concatenated and separated) of identifiers or names of taxon records and the associations of this occurrence to each of them.
countryCode	http://rs.tdwg.org/dwc/terms/countryCode; the standard code for the country in which the location of record occurs.
country	http://rs.tdwg.org/dwc/terms/country; the name of the country or major administrative unit in which the location of record occurs.
stateProvince	http://rs.tdwg.org/dwc/terms/stateProvince; the name of the next smaller administrative region than country.
locality	http://rs.tdwg.org/dwc/terms/locality; the specific description of the place.
kingdom	http://rs.tdwg.org/dwc/terms/kingdom; the full scientific name of the kingdom in which the taxon is classified.
phylum	http://rs.tdwg.org/dwc/terms/phylum; the full scientific name of the phylum or division in which the taxon is classified.
class	http://rs.tdwg.org/dwc/terms/class; the full scientific name of the class in which the taxon is classified.
order	http://rs.tdwg.org/dwc/terms/order; the full scientific name of the order in which the taxon is classified.
family	http://rs.tdwg.org/dwc/terms/family; the full scientific name of the family in which the taxon is classified.
genus	http://rs.tdwg.org/dwc/terms/genus; the full scientific name of the genus in which the taxon is classified.
specificEpithet	http://rs.tdwg.org/dwc/terms/specificEpithet; the name of the first or species epithet of the scientificName.
verbatimTaxonRank	http://rs.tdwg.org/dwc/terms/verbatimTaxonRank; the taxonomic rank of the most specific name (here - superfamily, as this option was absent in the mapping tool) used in the dataset.
type	http://purl.org/dc/elements/1.1/type; the nature or genre of the resource.

## Figures and Tables

**Table 1.** Helminth species identified to species level (ordered alphabetically within each phylum) recorded in the present study, their hosts and sites of infection: msc - mesocercariae of trematodes, mtc - metacercariae of trematodes, L3 - third-stage juveniles of nematodes.

**Parasite**	**Site of infection**	**Host species**	**Prevalence**, %	**Mean intensity**
**Acanthocephala: Palaeacanthocephala**				
*Acanthocephalusranae* (Schrank, 1788)	intestine	*B.bombina*	42.9	1.7
		*E.orbicularis*	3.7	2
		*R.temporaria*	47.8	4.2
		*R.arvalis*	33.3	1
		*P.ridibundus*	33.3	7.5
*Centrorhynchusspinosus* (Kaiser, 1893) (cystacanth)	body cavity	*H.orientalis*	20	3
*Pseudoacanthocephalusbufonis* (Shipley, 1903)	intestine	*B.bufo*	16.7	6
*Sphaerirostrispicae* (Rudolphi, 1819) (cystacanth)	body cavity & muscles	*Z.vivipara*	4.8	1
**Nematoda: Chromadorea**				
*Cosmocercacommutata* (Diesing, 1851)	rectum	*B.bufo*	16.7	33
	intestine & rectum	*B.viridis*	18.2	20.8
*Cosmocercaornata* (Dujardin, 1845)	intestine & rectum	*R.temporaria*	69.6	8.1
	intestine	*P.ridibundus*	16.7	1
*Entomelasdujardini* (Maupas, 1916)	lungs	*A.colchica*	66.7	6.5
*Entomelasentomelas* (Dujardin, 1845)	pharynx	*A.colchica*	50	8
*Falcaustraarmenica* Massino, 1924	intestine	*E.orbicularis*	22.2	8.8
*Icosiellaneglecta* (Diesing, 1851)	limb muscles	*P.ridibundus*	33.3	7.5
*Oswaldocruziabialata* (Molin, 1860)	intestine	*R.temporaria*	73.9	9.9
		*R.arvalis*	33.3	3
*Oswaldocruziaduboisi* Ben Slimane, Durette-Desset & Chabaud, 1993	intestine &stomach	*N.natrix*	8.7	8
*Oswaldocruziafiliformis* (Goeze, 1782)	intestine	*B.bufo*	100	25.2
*Oswaldocruzialacertica* Svitin, 2017	intestine	*L.agilis*	8.7	10.5
*Oswaldocruzialisnykiensis* Svitin, 2017	intestine	*A.colchica*	33.3	18.5
*Oswaldocruziaskrjabini* Travassos, 1937	intestine & stomach	*Z.vivipara*	23.8	1.3
*Oswaldocruziaukrainae* Iwanitzky, 1928	intestine &rectum	*B.viridis*	40.9	5.8
*Oxysomatiumbrevicaudatum* (Zeder, 1800)	lungs (L3)	*R.temporaria*	8.7	3
	intestine	*R.temporaria*	8.7	17.5
		*A.colchica*	16.7	4
	stomach	*N.natrix*	4.3	48
*Parapharyngodonszczerbaki* Radchenko & Sharpilo, 1975	intestine	*L.agilis*	4.3	123
*Rhabdiasbufonis* (Schrank, 1788)	lungs	*R.temporaria*	100	2.7
		*R.arvalis*	30.4	9.3
*Rhabdiasrubrovenosa* (Schneider, 1866)	lungs	*B.viridis*	22.7	8.2
*Rhabdiassphaerocephala* Goodey, 1924	lungs	*B.bufo*	91.7	7.2
*Serpentirhabdiasfuscovenosa* (Railliet, 1899)	lung	*N.natrix*	91.3	14.9
		*N.tessellata*	100	40.9
		*V.berus*	14.3	1
*Spiroxyscontortus* (Rudolphi, 1819)	stomach	*E.orbicularis*	85.2	22.2
	intestine	*N.natrix*	8.7	1
**Platyhelminthes: Trematoda**				
*Alariaalata* (Goeze, 1782) (msc)	body cavity	*N.natrix*	39.1	136.8
*Astiotremaemydis* Ejsmont, 1930	intestine	*E.orbicularis*	18.5	9.4
*Astiotremamonticellii* Stossich, 1904	intestine	*N.natrix*	43.5	146.5
		*N.tessellata*	28.6	5
*Diplodiscussubclavatus* (Pallas, 1760)	intestine	*H.orientalis*	40	1
	intestine & rectum	*P.ridibundus*	100	12
*Encyclometracolubrimurorum* (Rudolphi, 1819)	stomach	*N.natrix*	52.2	30.2
	body cavity (mtc)	*H.orientalis*	40	6
*Haematoloechusvariegatus* (Rudolphi, 1819)	lungs	*P.ridibundus*	14.3	1
		*B.bombina*	16.7	1
*Leptophallusnigrovenosus* (Bellingham, 1844)	oesophagus & intestine	*N.natrix*	13	17.3
*Macroderalongicollis* (Abildgaard, 1788)	lung	*N.natrix*	52.2	6
		*N.tessellata*	28.6	1.5
*Metaleptophallusgracillimus* (Luhe, 1909)	oesophagus & intestine	*N.natrix*	43.5	14.9
*Opisthioglypheranae* (Frolich, 1791)	intestine	*P.ridibundus*	83.3	27.4
		*R.temporaria*	4.3	1
		*B.bufo*	8.3	1
		*N.natrix*	4.3	3
*Paralepodermacloacicola* (Luhe, 1909)	intestine	*N.natrix*	52.2	26.7
		*N.tessellata*	42.9	29
*Plagiorchiselegans* (Rudolphi, 1802)	intestine	*L.agilis*	4.3	2
*Pleurogenesclaviger* (Rudolphi, 1819)	intestine	*R.temporaria*	4.3	1
		*B.bufo*	8.3	17
*Prosotocusconfusus* (Looss, 1894)	intestine	*P.ridibundus*	16.7	10
*Skrjabinoecessimilis* (Looss, 1899)	lungs	*R.arvalis*	33.3	3
*Strigeasphaerula* (Rudolphi, 1803) (mtc)	body cavity	*N.natrix*	52.2	125.9
		*N.tessellata*	28.6	42.5
*Telorchisassula* (Dujardin, 1845)	stomach	*N.natrix*	69.6	40.6
	intestine	*N.tessellata*	100	62.9
*Telorchisstossichi* Goldberger, 1911	intestine	*E.orbicularis*	33.3	118
*Tylodelphysexcavata* (Rudolphi, 1803) (mtc)	spine	*P.ridibundus*	33.3	34
**Platyhelminthes: Cestoda**				
*Ophiotaeniaeuropaea* Odening, 1963	intestine	*N.natrix*	82.6	6.8
		*N.tessellata*	71.4	5.8
**Platyhelminthes: Monogenea**				
*Polystomaintegerrimum* (Frolich, 1791)	urinary bladder	*R.temporaria*	39.1	1.8
*Polystomaviridis* Euzet, Combes & Batchvarov, 1974	urinary bladder	*B.viridis*	4.5	3
*Polystomoidesocellatum* (Rudolphi, 1819)	pharynx	*E.orbicularis*	25.9	1.4
